# Spiritual Care in the ICU: Perspectives of Dutch Intensivists, ICU Nurses, and Spiritual Caregivers

**DOI:** 10.1007/s10943-017-0457-2

**Published:** 2017-08-11

**Authors:** Suzan Willemse, Wim Smeets, Evert van Leeuwen, Loes Janssen, Norbert Foudraine

**Affiliations:** 1 Spiritual Care Department, VieCuri Medical Centre, P.O. Box 1926, 5900 BX Venlo, The Netherlands; 2 Department of Spiritual and Pastoral Care, Radboud University Nijmegen Medical Centre, Geert Grooteplein 21, EZ 6525 Nijmegen, The Netherlands; 3 Department of Ethics, Philosophy and History of Medicine, Radboud University Nijmegen Medical Centre, Geert Grooteplein 21, EZ 6525 Nijmegen, The Netherlands; 4 Department of Clinical Epidemiology, VieCuri Medical Centre, P.O. Box 1926, 5900 BX Venlo, The Netherlands; 5 Department of Critical Care, VieCuri Medical Centre, P.O. Box 1926, 5900 BX Venlo, The Netherlands

**Keywords:** Critical care, Intensive care, Spiritual care, Ethics, Existential and meaning of life issues, Quality of care, Quality of life

## Abstract

Since there are no scientific data available about the role of spiritual care (SC) in Dutch ICUs, the goal of this quantitative study was twofold: first, to map the role of SC as a part of daily adult ICU care in The Netherlands from the perspective of intensivists, ICU nurses, and spiritual caregivers and second, to identify similarities and differences among these three perspectives. This study is the quantitative part of a mixed methods approach. To conduct empirical quantitative cohort research, separate digital questionnaires were sent to three different participant groups in Dutch ICUs, namely intensivists, ICU nurses, and spiritual caregivers working in academic and general hospitals and one specialist oncology hospital. Overall, 487 participants of 85 hospitals (99 intensivists, 290 ICU nurses, and 98 spiritual caregivers) responded. The majority of all respondents (>70%) considered the positive effects of SC provision to patients and relatives: contribution to mental well-being, processing and channeling of emotions, and increased patient and family satisfaction. The three disciplines diverged in their perceptions of how SC is currently evolving in terms of information, assessment, and provision. Nationwide, SC is not implemented in daily ICU care. The majority of respondents, however, attached great importance to interdisciplinary collaboration. In their view SC contributes positively to the well-being of patients and relatives in the ICU. Further qualitative research into how patients and relatives experience SC in the ICU is required in order to implement and standardize SC as a scientifically based integral part of daily ICU care.

## Introduction

Intensive care units (ICUs) are dominated by technical medical resources and equipment; the monitoring of vital functions has become the cornerstone of the healing process. Patients and relatives frequently feel abandoned in this technologically advanced environment. As soon as an ICU patient wakes up, feelings of being alone with their doubts, worries, or even agony easily arise. A common feature of ICU patients is that they are no longer able to communicate in the usual way due to their illness or treatment (sedation and intubation). Such a situation requires special communication skills, patience and intuition from relatives and caregivers (Willemse 2017). Critical illness challenges ICU patients and their relatives to find meaning and to deal with suffering. Their religious background or non-religious spiritual-existential beliefs are valuable coping tools for adaption and redefining hope (Adolph et al. 2011).

Since end-of-life care is an important part of ICU care, prior studies (M. J. Balboni et al. 2013, 2014; T. Balboni et al. 2011) into end-of-life care offer valuable data for research into SC in the ICU. However, SC is not limited to terminal care and is an essential component of basic patient rights (Kortner 2009). Therefore, it is important to investigate the way in which SC is integrated into daily ICU care. Spiritual issues are not frequently discussed between patients or their relatives and ICU staff. Moreover, ICU healthcare workers (HCW: intensivists and ICU nurses) often leave the spiritual needs of patients and/or their relatives to the in-house spiritual caregiver or the patient’s own parish clergy. They consider them better qualified than themselves to deal with problems of this kind because of time-consuming schedules or lack of experience. (M. J. Balboni et al. 2013; Ford et al. 2014).

When ICU staff offer SC to patients, the offer is generally appreciated. However, when they decide themselves to administer SC, then its effectiveness is less. (Hughes et al. 2007).

Our study aim was twofold: first, to map the role of SC as part of daily adult ICU care in the Netherlands from the perspective of intensivists, ICU nurses, and spiritual caregivers; and second, to identify the similarities and differences between the perspectives of these three disciplines. Differences in perspectives provide guidance on future qualitative research into patients and relatives’ experiences with SC in order to implement SC as an integral part of daily intensive care.

## Methods

We used a mixed method to analyze separate questionnaires which were developed for intensivists, ICU nurses, and spiritual caregivers. Some questions were the same in all three questionnaires; others were specific to the discipline in question. The questionnaires consisted of 40 questions covering the following categories: (1) spiritual care provision; (2) HCW competency and time investment; (3) communication and interventions in the context of interdisciplinary cooperation; (4) SC effects, support, and implementation at the policy level. The design of the questionnaires was based on the intensivist’s (NF) and the spiritual caregiver’s (SW) personal work experience in the ICU as well as previous international research (Festic et al. 2012). The questionnaires were reviewed by various professionals, including methodologists, intensivists, ICU nurses, and spiritual caregivers. The digital questionnaires were sent to all ICUs in the Netherlands and were completed anonymously between May and October 2013. Only the type of hospital (university, teaching, or neither) and the regional distribution (rural or urban) could be traced. Reminders were sent and, if necessary, an additional request to complete the questionnaire was made by phone and mail, with the goal of having at least one fully completed questionnaire per discipline per hospital.

Questionnaire responses were analyzed using SPSS Statistics. Results were considered statistically significant when *p* < 0.05. Descriptive statistics and Chi-square tests were used to compare responses among groups. First of all, omnibus Chi-square tests were used to compare responses; those that differ significantly among any of the groups are highlighted in the tables (bold print). Secondly, in case of particularly important or notable findings, we performed additional Chi-square tests for between group comparisons, which are mentioned in the text of the result section.

Percentages presented in the text and tables sometimes exceed 100% because multiple answers were permitted for most questionnaire items. Not all answers are presented in the tables for the sake of brevity.

The study was performed in accordance with the ethical standards laid down in the 1964 Declaration of Helsinki and its later amendments (WMA 2013). As no patients were involved and all participants contributed anonymously, informed consent was waived by the ethical review board.

## Results

Ninety-two hospitals were approached to participate in the study, with an overall response rate of 92% (85 participating hospitals). Seven hospitals refrained from participating for the following reasons: no in-house spiritual care service (4), no in-house ICU (1), objections in principle (1), and long-term absence of a spiritual caregiver (1). The 85 participating hospitals could be classified as follows: 8 university hospitals, 44 teaching hospitals (including one specialized cancer center), and 33 non-teaching hospitals.

Together, the 85 hospitals yielded 487 respondents. In terms of disciplines, 99 intensivists, 290 ICU nurses, and 98 spiritual caregivers participated from 66 hospitals (78%), 77 hospitals (91%), and 79 hospitals (93%), respectively (Table 1).

**Table 1:** Participant characteristics

Characteristics	All respondents (*n* = 487) N%	Intensivists (*n* = 99) N%	ICU nurses (*n* = 290) N%	Spiritual caregivers (*n* = 98) N%
Gender				
Male	204 (41.9)	64 (64.6)	85 (29.3)	55 (56.1)
Female	280 (57.5)	35 (35.4)	203 (70.0)	42 (42.9)
Missing	3 (0.6)	–	2 (0.7)	1 (1.0)
Age (years)				
Mean (SD)	46.1 (9.6)	44.8 (7.0)	43.9 (9.6)	53.6 (7.6)
Missing	2	1	1	2
Years of experience				
0–3 years	46 (9.4)	10 (10.1)	25 (8.6)	11 (11.2)
3–10 years	139 (28.5)	52 (52.5)	70 (24.1)	17 (17.3)
>10 years	299 (61.4)	37 (37.4)	194 (66.9)	68 (69.4)
Missing	3 (0.6)	–	1 (0.3)	2 (2.0)
Work setting				
Academic hospital	77 (15.8)	19 (19.2)	49 (16.9)	9 (9.2)
Community-based teaching hospital	196 (40.3)	38 (38.4)	119 (41.0)	39 (39.8)
Other teaching hospital	69 (14.2)	19 (19.2)	31 (10.7)	20 (20.4)
Non-teaching hospital	142 (29.2)	23 (23.2)	89 (30.7)	30 (30.6)
Missing	2 (0.4)	–	2 (0.7)	–
Number of hospitals	85 (92.3)	66 (77.6)	77 (90.5)	79 (93.0)

The item response rate was high: 95% of the substantive questions were answered by 92% of the intensivists, 95% of the ICU nurses, and 92% of the spiritual caregivers.

### Spiritual Care Provision

In 92% of all participating hospitals, a spiritual caregiver provided SC support in the ICU. More than 80% of all respondents considered patients’ philosophy of life and their spiritual background important or very important in how they cope with their illness. It should be mentioned that the respondents were free to define “philosophy of life/spirituality” as they wished.

The results showed that 13% of the intensivists and 12% of the ICU nurses indicated that patients and/or their relatives had shared with them their philosophy of life regarding spirituality “at least most of the time.” In contrast, 76% of the spiritual caregivers indicated that patients talked about this topic with them (*p* < 0.001).

Patients and their relatives were informed about the availability of SC in more than one way. Their awareness of this availability was largely attained through the ICU nurses and by means of a brochure. Still, the respondents estimated that 10% of all ICU patients and/or relatives were not offered any information about the possibility of SC. Significant differences were noted between the three disciplines in terms of their provision of information. Provision of information about the availability of SC to patients and/or relatives by intensivists was twice as high according to intensivists themselves (35%) than according to ICU nurses (15%) and spiritual caregivers (16%) (*p* < 0.001). There was a significant difference in the perception of responsibility for calling the spiritual caregiver after notifying the patient of the availability of SC. Intensivists surmised that they were responsible in 71% of cases; however, ICU nurses thought that intensivists were responsible in only 41% of all cases (*p* < 0.001). The intensivists’ and ICU nurses’ estimates of involving a spiritual caregiver at the intensivists’ request through an ICU nurse differed considerably: 68 versus 24%, *p* < 0.001, Table 2). Furthermore, one out of three respondents reported that SC was provided through the spiritual caregiver’s initiative only.

**Table 2:** Spiritual care provision

Spiritual care provision	All respondents (*n* = 487) N%	Intensivists (*n* = 99) N%	ICU nurses (*n* = 290) N%	Spiritual caregivers (*n* = 98) N%	***p*** **/pr**
The role of the philosophy of life/spirituality of the patient in the way the patient copes with his/her illness **(scores** **≥** 4)*	423 (86.8)	81 (81.8)	254 (87.5)	88 (89.7)	*p*
Provision information SC by intensivists	95 (19.5)	**35 (35)**	**44 (15)**	**16 (16)**	pr
SC called at intensivist’s request	256 (52.6)	**71 (71)**	**118 (40.7)**	**67 (68)**	pr
SC called at intensivist’s request through ICU nurse	137 (35.2)	**67 (67.7)**	**70 (24.1)**	N/A	pr
Not offered information on spiritual care	62 (12.7)	13 (13.1)	40 (13.8)	9 (9.2)	pr
Own initiative spiritual caregiver	182 (37.4)	**27 (27.3)**	**110 (37.9)**	**45 (45.9)**	pr
Oral request for SC	295 (75.8)	**65 (66)**	**230 (79)**	N/A	pr
Telephone request for SC	150 (38.6)	**19 (19)**	**131 (45)**	N/A	pr
Written request for SC	24 (6.2)	4 (4)	20 (7)	N/A	pr
**Important indicators for HCW to consult a spiritual caregiver (scores** **≥** 4)*					
Questions regarding the meaning of illness and existence*	352 (90.4)	89 (89.9)	263 (90.7)	N/A	pr
Lack of community support*	311 (79.9)	75 (76.5)	236 (83.1)	N/A	pr
Problems with a certain image of God*	290 (74.6)	72 (72.7)	218 (75.1)	N/A	pr
Ethical questions concerning withdrawing treatment*	256 (65.8)	**57 (57.6)**	**199 (68.6)**	N/A	pr
Problems with religious customs*	237 (62.4)	60 (60.6)	177 (70)	N/A	pr
Despondency*	223 (57.3)	49 (49.5)	174 (60)	N/A	pr
Problems with rituals*	207 (53.2)	47 (47.5)	160 (55.2)	N/A	pr
**Important actions (scores** **≥** 4)* by HCW in case of questions regarding the meaning of illness and existence of patients					
Identify questions regarding the meaning of illness and existence*	354 (91.0)	92 (92.9)	262 (90.3)	N/A	*p*
Explore these questions*	345 (88.7)	86 (86.9)	259 (89.3)	N/A	*p*
Guide the patient*	310 (79.7)	71 (71.7)	239 (82.4)	N/A	*p*
Refer the patient to other professionals*	365 (93.8)	87 (87.9)	278 (95.9)	N/A	*p*
Never signaled an existential question in the last month	99 (20.3)	**15 (15.2)**	**77 (26.6)**	**7 (7.1)**	pr
Signaled only one existential question in the last month	90 (18.5)	**11 (11.1)**	**74 (25.5)**	**5 (5.1)**	pr
Patient’s sharing of philosophy of life/spirituality at least most of the time	122 (25)	**13 (13.1)**	**35 (12.1)**	**74 (75.5)**	pr

* 5-point Likert scale: 1 = very unimportant, 2 = not important, 3 = neutral, 4 = important, 5 = very important

*p* SC referring to patient, *pr* SC referring to patient and/or relatives, *HCW* healthcare workers responses in bold print indicate a significant difference (*p* < 0.05) among the respondent groups

Table 2 shows that HCW most often consult a spiritual caregiver through an oral request and less than 7% of HCW made a written request for SC despite legislation requiring documentation of all aspects of health care delivered to patients. ICU nurses reported more frequently making requests by telephone compared to intensivists (45% vs 19%, *P* < 0.001, Table 2). According to 94% of all respondents, involvement of the spiritual caregiver takes place “at the patient’s request.”

All respondents took care of active requests for SC from patients and/or relatives, in particular questions regarding the meaning of illness and existence. These questions are the most important indicators for consulting a spiritual caregiver. Intensivists and ICU nurses considered dealing with these ethical questions in relation to an ICU patient an important part of the spiritual caregiver’s job (77 and 85%, respectively). Ninety percent of HCW indicated that in the case of questions regarding the meaning of illness and existence their professional actions are important or very important. (Table 2). However, 15% of the intensivists and 27% of the ICU nurses indicated that these questions were “never” asked in the month prior to completing the questionnaire (*p* < 0.001). According to the majority of HCW (78%), patients and relatives declined the offer of spiritual care primarily because they had “no need for spiritual care,” while according to the majority of spiritual caregivers (81%), “insufficient knowledge of spiritual care availability” was the reason for declining the offer. Not only patients and their relatives have access to SC. HCW can ask for SC for themselves in case of emotional distress. One-third of intensivists (29%) and ICU nurses (36%) felt it was important to be able to rely on a spiritual caregiver for themselves after the death of a particular patient. The results show that 16% of HCW consider collegial support given by the spiritual caregiver to ICU staff as part of SC provision. In contrast, 49% of the spiritual caregivers consider collegial support to ICU staff as one of their tasks (*p* < 0.001).

### HCW Competency and Time Investment

Table 3 provides an overview of HCW competency and time investment related to patients’ spiritual needs. As can be seen, 66% of HCW consider themselves capable of discussing existential questions and the meaning of illness. However, 90% of HCW indicated that the spiritual caregiver was the most appropriate professional to explore patients’ needs for SC.

**Table 3:** HCW competency and time investment

HCW competency and time investment	All respondents (*n* = 487) N%	Intensivists (*n* = 99) N%	ICU nurses (*n* = 290) N%	Spiritual caregivers (*n* = 98) N%
Competent to address existential questions	257 (66)	69 (69.7)	188 (64.8)	N/A
Intensivist should answer existential questions	209 (53.7)	**71 (71.1)**	**138 (47.6)**	N/A
ICU nurse should answer existential questions	232 (59.6)	56 (56.6)	176 (60.7)	N/A
Spiritual caregiver should answer existential questions	357 (91.7)	89 (89.9)	268 (92.4)	N/A
HCW needs ≤ 30 min to address existential questions him/herself	278 (71.5)	**74 (74.7)**	**254 (89.1)**	N/A
Insufficient time to address questions	107 (22.0)	**32 (32.3)**	**59 (20.3)**	**16 (16.3)**
Calls are answered by the spiritual caregiver within 1–2 h primarily for				
Conducting talks	461 (94.6)	89 (89.9)	277 (95.5)	95 (96.9)
Presence with patient and/or relatives	377 (77.4)	**84 (84.8)**	**212 (73.1)**	**81 (82.7)**
Working with rituals	366 (75.1)	**77 (77.8)**	**202 (69.7**)	**87 (88.8)**
Collegial support by the spiritual caregiver to ICU staff	109 (22.3)	**18 (18.2)**	**43 (14.8)**	**48 (49)**
and in difficult situations*				
Death expected soon	377 (77.4)	81 (81.8)	228 (78.6)	68 (69.4)
Withdrawal of treatment	344 (70.6)	**78 (78.8)**	**208 (71.7)**	**58 (59.2)**
A life-threatening situation	334 (68.6)	64 (64.6)	202 (69.7)	68 (69.4)
Longer than average length of stay	333 (68.4)	72 (72.7)	202 (69.7)	59 (60.2)
Problems in the relations between patient and relatives	257 (52.8)	**67 (67.7)**	**146 (50.3)**	**44 (44.9)**
Organ donation	216 (44.4)	**50 (50.5)**	**150 (51.7)**	**16 (16.3)**

* >1 time a monthResponses in bold print indicate a significant difference (*p* < 0.05) among the respondent groups

The extent to which intensivists thought that they had to answer existential questions (71%) and the extent to which ICU nurses indicated that intensivists should answer these questions (48%) differed significantly (*p* < 0.001). More than 74% of the intensivists and ICU nurses thought that they would need 20–30 min to discuss existential questions. Reasons given for having insufficient time to explore these questions were reported as “too many other tasks” (61% of intensivists), “patient complexity” (76% of ICU nurses), and too few permanent positions” (24% of spiritual caregivers). In general, spiritual caregivers answered calls within 1–2 h, with an average of 3 consultations at a mean duration of 15–30 min each. According to all three disciplines, SC primarily takes shape by conducting talks, as well as through presence with patient and/or relatives and working with rituals. Spiritual caregiver involvement at HCW request in explicit situations like withdrawal of treatment, problems in the relations between patient and relatives, and organ donation was not rated as highly by spiritual caregivers as it was by HCW (Table 3).

### Communication and Interventions in the Context of Interdisciplinary Cooperation

Table 4 shows that SC provided by a spiritual caregiver is not yet embedded in daily ICU care. Forty-six percent of HCW indicated that cooperation with the spiritual caregiver took place on demand. The intensivists indicated that spiritual caregivers participated in ethical considerations twice as often as in multidisciplinary rounds (MDRs). Spiritual caregivers mainly reported their findings in a patient file. They reported orally to ICU nurses twice as often as to intensivists. Spiritual caregivers refrained sometimes (11%) from reporting for reasons of confidentiality.

**Table 4:** Interdisciplinary collaboration: SC effects, support, and implementation at the policy level

Interdisciplinary collaboration SC effects, support, and implementation of SC at the policy level	All respondents (*n* = 487) N%	Intensivists (*n* = 99) N%	ICU nurses (*n* = 290) N%	Spiritual caregivers (*n* = 98) N%
Interdisciplinary collaboration				
On demand	225 (46.2)	45 (45.5)	139 (47.9)	41(41.8)
SC implemented in multidisciplinary care	99 (20.3)	**22 (22.2)**	**42 (14.5)**	**35 (35.7)**
No interdisciplinary collaboration yet	60 (12.3)	**16 (16.2)**	**41 (14.1)**	**3 (3.1)**
Participation of the spiritual caregiver in structural forms				
No consultation forms	256 (52.5)	43 (43.4)	159 (54.8)	54 (55.1)
Ethical considerations	161 (33)	**44 (44.4)**	**85 (29.3)**	**32 (32.7)**
MDRs (multidisciplinary rounds)	92 (18.8)	20 (20.2)	47 (16.2)	25(25.5)
Team meetings	29 (5.9)	10 (10.1)	15 (5.2)	4 (4.1)
Conditions for interdisciplinary collaboration				
Sufficient knowledge of SC provision	371 (76.1)	**82 (82.8)**	**202 (69.7)**	**87 (88.8)**
HCW attention to indicators of spiritual needs	360 (73.9)	**73 (73.7)**	**195 (67.2)**	**92 (93.9)**
Protocol for SC	328 (67.3)	**58 (58.6)**	**193 (66.6)**	**76 (77.6)**
Transparent approach of spiritual caregiver	281 (57.7)	**49 (49.5)**	**154 (53.1)**	**78 (79.6)**
MDRs (multidisciplinary rounds)	197 (40.4)	**32 (32.3)**	**107 (36.9)**	**58 (59.2)**
Attention to HCW’s emotional problems	182 (37.3)	**31 (31.3)**	**93 (32.1)**	**58 (59.2)**
Anchoring SC at the policy level	169 (34.7)	**25 (25.3)**	**70 (24.1)**	**74 (75.5)**
SC effects (scores ≥ 4)*				
Positive contribution to mental well-being	355 (72.8)	**66 (66.7)**	**203 (70)**	**86 (87.8)**
Processing and channeling emotions	328 (67.3)	66 (66.7)	188 (64.8)	74 (75.5)
Increased patient and family satisfaction	326 (66.9)	61 (61.7)	195 (67.3)	70 (71.5)
Phenomena encountered with the patient and relatives when SC is provided (scores ≥ 4)*				
Despair as a result of not being in control	222 (45.5)	49 (49.5)	123 (42.4)	50 (51)
Vain search for hope and perspective	214 (43.9)	47 (47.5)	131 (45.2)	36 (36.8)
Questions about making choices regarding treatment in the light of moral conviction	178 (36.5)	**44 (44.5)**	**104 (35.8)**	**30 (30.6)**
Support and implementation of SC at the policy level				
ICU management	**325 (66.7)**	**72 (72.7)**	**176 (60.7)**	**77 (78.6)**
The boards of the participating hospitals	**372(76.3)**	**74(74.7)**	**211(72.8)**	**87(88.8)**

* 5-point Likert Scale: 1 = never, 2 = usually not, 3 = sometimes, 4 = usually, 5 = always

Responses in bold print indicate a significant difference (*p* < 0.05) among the respondent groups

All three disciplines attached great importance to interdisciplinary collaboration and regarded it as an important condition for spiritual caregiver intervention. In addition, according to the respondents, the following conditions in particular are necessary for integrated cooperation with the spiritual caregiver: (1) sufficient knowledge of SC provision among the various disciplines working in the ICU, (2) HCW’s attention to indicators of spiritual needs, and (3) binding agreements with respect to SC in accordance with protocols (Table 4).

### SC Effects, Support, and Implementation at the Policy Level

Since there is no validated instrument to measure the effects of spiritual care in ICUs, all three disciplines were asked to answer questions about their experiences concerning these effects on patients and their relatives. The effects of SC in the ICU were usually positively experienced by >70% of all three disciplines. Table 4 shows that those effects were (1) positive contribution to mental well-being, (2) processing and channeling emotions, and (3) increased patient and family satisfaction. The table also shows the phenomena encountered with the patient and the patient’s relatives when spiritual care is provided, during or after SC provision.

A majority of all respondents believed that ICU management supported SC, which might have been reflected in the ICU policy documentation. Respondents showed high scores (>72%) in terms of SC support on the boards of the participating hospitals at the policy level. (Table 4).

## Discussion

This study’s aim was to map the role of SC as part of daily ICU care in Dutch adult ICUs from the perspective of intensivists, ICU nurses, and spiritual caregivers. Furthermore, data were collected to identify the similarities and differences among those three perspectives. According to the majority of respondents from all three perspectives, patients and their relatives benefit from SC with respect to quality of care and quality of life. This finding corresponds to the results of previous international studies (Gries et al. 2008, 2010; Johnson et al. 2014; Kirchhoff & Faas 2007; Kirchhoff et al. 2008; Rosik & Soria 2012; Wall et al. 2007). How patients and relatives experience SC in the ICU is an important target for further qualitative research (Kross et al. 2009; Wahlin et al. 2009). Moreover, the vast majority of respondents considered the patient’s philosophy of life and spirituality most important in how he/she copes with illness. These results match those observed in an earlier study (Delgado-Guay et al. 2011).

In agreement with past research, the majority of Dutch HCW think that the spiritual caregiver is the primary professional to explore questions of existential meaning and ethical issues (Curtis & Vincent 2010; Jensen et al. 2013).

Although almost all Dutch ICUs have at least one spiritual caregiver, hardly any of these ICUs have standardized SC methods to incorporate the assessments of the spiritual needs of patients and/or their relatives in the ICU. Earlier studies confirm the importance of such standardization (Benito et al. 2014; Hughes et al. 2007; Smeets et al. 2011). Regarding SC information, assessment, and provision, the results show significant differences among the perspectives of intensivists, ICU nurses, and spiritual caregivers. In relation to SC provision, a discrepancy emerged between intensivists’ and ICU nurses’ views of when they take the initiative to call in the spiritual caregiver. ICU nurses spend much more time with the ICU patients than intensivists, and probably feel more involved with the patient. This might explain the differences observed. In concordance, the frequency of spiritual caregivers reporting to ICU nurses after visiting the patient and/or relatives is twice as high as for intensivists (James et al. 2011; Lundberg & Kerdonfag 2010).

Could it be that intensivists are not always aware of ICU nurses’ taking the initiative to involve a spiritual caregiver, or that spiritual caregivers feel more comfortable reporting to ICU nurses? This observation calls for improvement of internal communication among ICU staff and is in accordance with documented differences among HCW in an earlier study regarding spiritual assessment in end-of-life care (Festic et al. 2012; Schenker et al. 2012). Other studies similarly show the importance of signaling the need for SC support for patients and relatives (Ho et al. 2011; Penrod et al. 2012).

The current study shows that in cases in which SC is offered by the HCW or spiritual caregiver and subsequently declined, patients and relatives decline the offer of SC primarily because of having “no need for SC.” Spiritual caregivers consider “insufficient knowledge of SC availability” the main reason for declining that offer. Education of HCW by spiritual caregivers could contribute to the improvement of communication concerning SC provision (Ford et al. 2014; Gordon et al. 2012; Puchalski et al. 2014; Schaefer & Block 2009). Moreover, most HCW are not aware of how SC can contribute to their own mental well-being (Guthrie 2014; Poncet et al. 2007; St Ledger et al. 2013; Wahlin et al. 2010). This may explain the significant difference between the response of spiritual caregivers and the HCW in relation to collegial support by the spiritual caregiver to ICU staff. The majority of respondents regarded multidisciplinary cooperation in the ICU as important. International studies confirm the importance of integrated SC in the ICU (Cook & Rocker 2014; Handzo et al. 2014; Ho et al. 2011; Hughes et al. 2007; Loscalzo 2008; Truog et al. 2008). The current study shows that all HCW regard sufficient knowledge of SC provision and attention to signaling of patients’ spiritual needs as important conditions for interdisciplinary collaboration. Moreover, only one out of three spiritual caregivers stated that SC was implemented in multidisciplinary care. Apparently, spiritual care is not an integral part of daily intensive care in Dutch ICUs. This may explain the significant differences reflected in Table 4.

The majority of respondents indicated that they had enough time to explore questions regarding the meaning of illness and existence. However, in the case of insufficient time, “too many other tasks” and “patient complexity” were the main barriers mentioned. Barriers HCW encountered in relation to SC support mentioned in earlier studies are “insufficient time” and “insufficient education” (M. J. Balboni et al. 2014; Gordon et al. 2012; Ronaldson et al. 2012). All these barriers have important implications for the development of the role of SC in the ICU.

As concluded in previous studies, it is recommended that spiritual caregivers provide evidence-based SC to improve the quality of care, following the example set by physicians who routinely provide evidence-based care (Curtis & White 2008; Fitchett 2011; Fitchett et al. 2014; Handzo et al. 2014; Kalish 2012). It is encouraging that a vast majority of spiritual caregivers have a positive attitude toward examining patient, family, and ICU staff satisfaction (Fitchett et al. 2014; Handzo et al. 2014).

Finally, according to three-quarters of the respondents, the boards of the participating hospitals supported SC at policy level. To secure this support, further scientific research into SC in hospitals is of vital importance.

We are aware that the results of our study are susceptible to selection bias, especially as the number of responding intensivists and ICU nurses was a relatively low fraction of all HCW in the ICUs. However, every respondent was asked to complete the questionnaire in light of daily ICU practice as much as possible and not only based on personal experience/opinions. Since 85 out of 92 hospitals participated, we think this study gives a representative overview of SC in ICUs in the Netherlands, despite the aforementioned restriction.

## Conclusions

This study shows that SC is not yet an integrated part of daily ICU care at a national level, despite the finding that the majority of intensivists, ICU nurses, and spiritual caregivers think SC contributes positively to the well-being of patients and relatives in the ICU.

Additional findings included other similarities, but also differences in experiences with SC in the ICU from the perspectives of health care workers (HCW: intensivists and ICU nurses) and spiritual caregivers, and barriers that both HCW and spiritual caregivers encounter in ICU care. Moreover, this study points toward improvement of internal communication and interdisciplinary collaboration, expansion of knowledge of SC provision, and provision of evidence-based SC practice.

Our findings call for the second part of the mixed methods approach, namely qualitative research into the experiences of patients and their relatives in relation to SC in the ICU.

Since the mixed methods approach compares, validates, and corroborates the data of quantitative and qualitative research, it will give an insight into how the implementation and subsequent improvement of SC in daily ICU care can be realized, with the aim of improving quality of life for ICU patients and their relatives.
